# B-Cell Lymphoma of the Thoracic Spine Presenting with Spinal Cord Pressure Syndrome

**DOI:** 10.4021/jocmr2010.02.258w

**Published:** 2010-02-26

**Authors:** Mazen Sanoufa, Mohammad Sami Walid, Talat Parveen

**Affiliations:** a Medical Center of Central Georgia, Macon, GA, USA

## To the Editor:

B-cell lymphomas are the most common type (85%) of non-Hodgkin lymphomas [1]. Diffuse large B-cell lymphoma accounts for a third of these tumors and occurs mostly in older people [1]. Non-Hodgkin's lymphomas arising in the spinal cord are extremely rare. Spinal cord compression as the initial manifestation of non-Hodgkin's lymphoma is rare and may occur in advanced stages of the disease [1-3]. It usually looks isointense or low signal (relative to the spinal cord) on T1-weighted images and high signal on T2-weighted images [4]. Contrast enhancement is usually intense and homogeneous [4].

A 70-year-old African-American male presented to the emergency room complaining of bilateral mild lower extremity weakness for 2 days. The patient could not bear weight on his legs when he tried to get out of bed in the last two mornings. He also felt numb in both legs. Since then, he has been incontinent of urine but not feces. He denied any back pain or trauma. He also had no weakness or numbness in the upper extremities.

His past medical history included chronic obstructive pulmonary disease and aortic regurgitation. He was on Zithromax and Prednisone at Home. He denied using alcohol, tobacco or elicit drugs. The patient had some wheezing and cough in addition to mild abdominal pain. On neurological exam, the patient had weakness in the lower extremities. The strength at the hip and knee was 2/5 on the right side and 3/5 on the left side and 1/5 at the ankle on both sides. Deep tendon reflexes were 2+ in the biceps and triceps symmetrically. His right knee jerk was 1+ and left knee jerk 2+ and both ankle jerks were abscent. He also had absence of position sense in both of his ankles and feet. He had a pinprick sensation at the nipple level (T4) both anteriorly and posteriorly.

**Figure 1:**
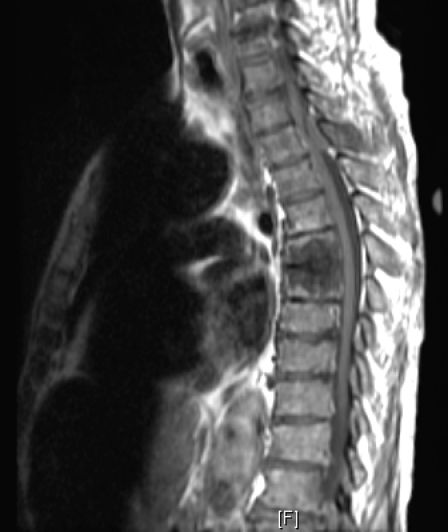
MRI T1.

**Figure 2:**
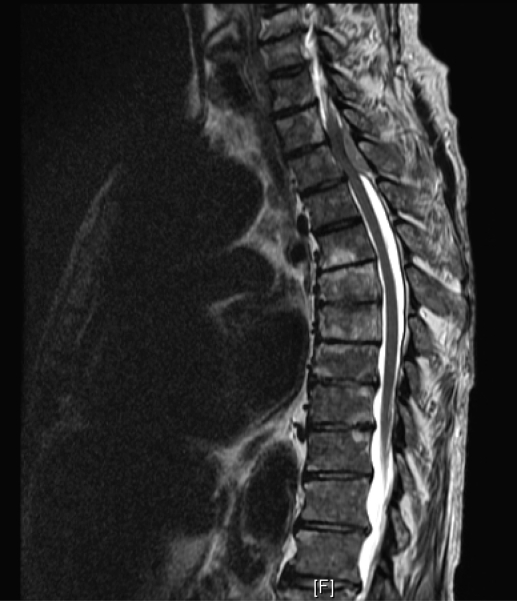
MRI T2.

**Figure 3:**
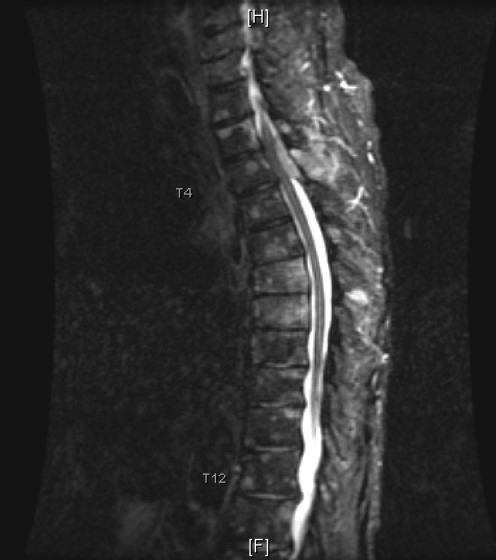
MRI with contrast.

The initial diagnosis was acute spinal cord syndrome at T4 and the patient was put on Decadron. Thoracic MRI showed an epidural soft tissue mass at the T3, T4 and T5 levels extending from the posterior aspect of the spinal canal causing moderate encroachment upon the spinal cord at T4 with diffuse metastatic disease involving the cervical and lumbar vertebral bodies (Fig. 1-3). After consulting with the patient it was decided to proceed with T3, T4, T5 decompressive laminectomies and excision of the tumor from the epidural space under microscope.

**Figure 4:**
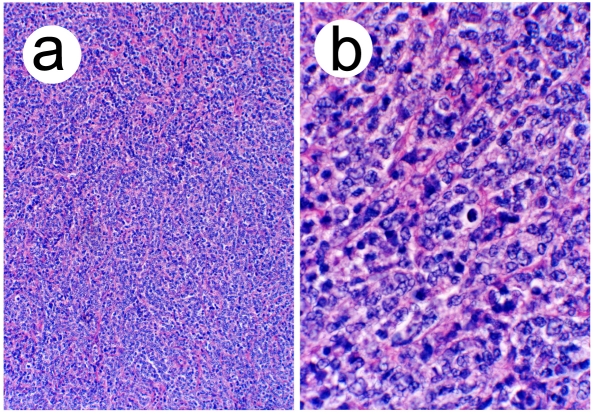
(a) Low power view showing diffuse proliferation of lymphoid cells (hematoxylin-eosin, original magnification X40). (b) High power view demonstrating nuclear features (hematoxylin-eosin, original magnification X400).

**Figure 5:**
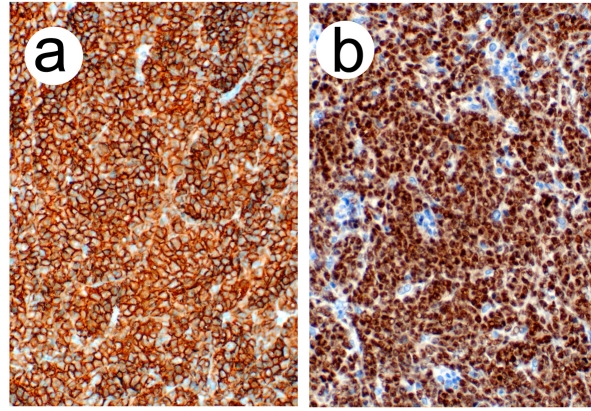
(a) CD 20 positive lymphoid cells (immunoperoxidase stain, original magnification X200). (b) PAX5 positive lymphoid cells (immunoperoxidase stain, original magnification X200).

Microscopic examination revealed a diffuse proliferation of intermediate-to-large lymphocytes with vesicular nuclei, nuclear irregularities, occasional distinct nucleoli and scant cytoplasm. The mitotic rate was brisk. The stroma showed sclerosis and vascular proliferation. Immunoperoxidase stains revealed that the neoplastic cells were weakly positive for leukocyte common antigen (CD45), strongly positive for B-cell markers (CD20, PAX5, CD79a, MUM-1+ and BCL-6+) and negative for T-cell markers (CD3 and CD5). They were also negative for BCL-2, CD10, cyclin D1, CD138, CD34, ALK-1 and CD30. Additionally, they were negative for carcinoma, neuroendocrine and neural markers. The findings were thus consistent with a diffuse large B-cell lymphoma (Fig. 4-5).

The patient underwent chemotherapy and radiation therapy but he expired within several months after diagnosis.

## References

[1] Chahal S, Lagera JE, Ryder J, Kleinschmidt-DeMasters BK (2003). Hematological neoplasms with first presentation as spinal cord compression syndromes: a 10-year retrospective series and review of the literature. Clin Neuropathol.

[2] Chiodo A (2007). Spinal cord injury caused by epidural B-cell lymphoma: report of two cases. J Spinal Cord Med.

[3] Perry JR, Deodhare SS, Bilbao JM, Murray D, Muller P (1993). The significance of spinal cord compression as the initial manifestation of lymphoma. Neurosurgery.

[4] Boukobza M, Mazel C, Touboul E (1996). Primary vertebral and spinal epidural non-Hodgkin's lymphoma with spinal cord compression. Neuroradiology.

